# Assessment of Modified Citrus Pectin’s Effects on Dementia in the Scopolamine-Induced Alzheimer’s Model in Adult Male Wistar Rats

**DOI:** 10.3390/cimb46120832

**Published:** 2024-12-11

**Authors:** Jale Akgöl, Özden Kutlay, Arzu Keskin Aktan, Fatma Fırat

**Affiliations:** 1 Department of Medical Pharmacology, Faculty of Medicine, Afyonkarahisar Health Sciences University, 03030 Afyonkarahisar, Turkey; 2 Department of Physiology, Faculty of Medicine, Afyonkarahisar Health Sciences University, 03030 Afyonkarahisar, Turkey; ozden.kutlay@afsu.edu.tr (Ö.K.); arzu.aktan@afsu.edu.tr (A.K.A.); 3 Department of Histology and Embryology, Faculty of Medicine, Afyonkarahisar Health Sciences University, 03030 Afyonkarahisar, Turkey; fatma.firat@afsu.edu.tr

**Keywords:** galectin-3, Alzheimer’s, modified citrus pectin, dementia, antioxidant effect, anti-inflammatory effect

## Abstract

Modified citrus pectin (MCP) modulates galectin-3, a key player in neuroinflammation linked to Alzheimer’s disease. By inhibiting galectin-3, MCP reduces the brain’s inflammatory response and may alleviate cognitive decline. This study examines MCP’s impact on neuroinflammation, cognitive function, and its role in galectin-3 inhibition in a dementia model. Male Wistar rats were assigned to four groups: control (*n* = 6), scopolamine (SCP) *(n* = 7), SCP + MCP (*n* = 7), and MCP only (*n* = 7). MCP was administered orally at 100 mg/kg/day via drinking water for six weeks. SCP was injected intraperitoneally at 1 mg/kg for seven days to induce an Alzheimer’s-type dementia model. The researchers assessed cognitive performance through the Morris Water Maze (MWM) test. After behavioral tests, blood and brain tissues, including the hippocampus, were collected and stored at −80 °C for analysis. Immunohistochemistry was used to evaluate superoxide dismutase (SOD) activity, malondialdehyde (MDA) levels, brain-derived neurotrophic factor (BDNF), and inflammatory markers (IL-1β, IL-6, TNF-α, and galectin-3). The data were analyzed with SPSS 22. SCP treatment increased lipid peroxidation (MDA) and elevated inflammatory markers (TNF-α, IL-6, and galectin-3), while reducing BDNF and impairing spatial memory. Co-administering MCP with SCP significantly reduced TNF-α, IL-6, and galectin-3 levels; increased BDNF; and improved memory performance. Although MCP did not lower MDA levels, it boosted SOD activity, suggesting antioxidant effects. Modified citrus pectin (MCP) alleviated cognitive impairments and reduced neuroinflammation in Alzheimer’s-type dementia by inhibiting galectin-3. MCP also exhibited antioxidant potential, underscoring its therapeutic promise for neurodegenerative diseases.

## 1. Introduction

Alzheimer’s type dementia is a neurological condition characterized by a gradual loss of memory and a long-term decline in executive functions, socio-behavioral skills, and visuospatial abilities. In Alzheimer’s disease, the hippocampus is among the first regions in the brain to be affected [1]. The primary pathogenic hallmarks of Alzheimer’s disease, a progressive neurodegenerative disorder, include the formation of extracellular amyloid beta (Aβ) peptide aggregates, fibrillary tangles of intraneuronal tau, and the activation of microglia [2]. Although the exact cause of Alzheimer’s disease remains unclear, several theories have been proposed to explain its mechanisms. Neuroinflammation is increasingly recognized as a critical factor in Alzheimer’s pathogenesis. Microglia adhering to pathological Aβ through their receptors are activated, releasing pro-inflammatory cytokines and inducing functional and structural changes that lead to neuronal degeneration in the central nervous system [3,4].

Galectin-3 (Gal-3) plays a multifaceted role in both extracellular and intracellular environments, influencing processes such as cell adhesion, survival, proliferation, and inflammation. Research in humans and animals demonstrates that Gal-3 significantly modulates microglial activity and neuroinflammation [5]. Studies reveal that the levels of Gal-3, a member of the galectin protein family, are elevated in Alzheimer’s disease. In Gal-3 knockout mice, a decrease in Aβ oligomerization correlates with improved cognitive test performance [6]. Silencing Gal-3 in monocyte-derived dendritic cells reduces the levels of pro-inflammatory mediators, including interleukin-1 beta (IL-1β), interleukin-6 (IL-6), and tumor necrosis factor-alpha (TNF-α). Gal-3 also regulates acute and chronic inflammation as well as tissue fibrogenesis [7].

Gal-3 is pivotal in the etiology of neuroinflammatory and neurodegenerative conditions, including multiple sclerosis, Alzheimer’s disease, Parkinson’s disease, and Huntington’s disease. In Alzheimer’s disease, its involvement in neuroinflammation, Aβ accumulation, and signaling dysregulation make it a promising target for therapeutic interventions aimed at slowing disease progression and reducing neuronal damage. Gal-3 is considered a novel therapeutic target in combating Alzheimer’s disease. Anti-galectin treatments represent a potential new avenue in this regard [8]. Gal-3 also negatively regulates hippocampus-dependent memory formation. Evidence shows that Gal-3 influences inflammatory pathways and cognitive function through its pro-inflammatory role. Based on this evidence, this study hypothesizes that modified citrus pectin (MCP), a Gal-3 antagonist, may offer therapeutic benefits for dementia [9,10]. Recent years have seen a significant shift in our understanding of microglial activation. Galectin-3 (Gal-3) plays a critical role in the polarization of microglia under pathological conditions and can modulate immune responses. In the central nervous system (CNS), Gal-3 interacts with proteins such as Toll-like receptors (TLRs), which in turn alter microglial responses. Through its interaction with TLR4, Gal-3 can either enhance or suppress microglial inflammatory reactions. Additionally, Gal-3 directs microglia to initiate phagocytic responses for clearing waste and nutrients. In Alzheimer’s disease (AD), Gal-3 is linked to microglial activation around amyloid-β (Aβ) plaques. Elevated levels of Gal-3 are observed in the early stages of AD, influencing the microglial immune response. The interaction between Gal-3 and TREM2 further supports the role of microglia in AD. Studies show that inhibiting Gal-3 reduces microglial activation and slows the progression of AD, making Gal-3 a promising therapeutic target for neurodegenerative diseases such as Alzheimer’s [11].

Modified citrus pectin (MCP) is a form of pectin that has been chemically modified for better absorption in the digestive tract [12]. MCP can tightly bind with Gal-3 by recognizing its carbohydrate recognition domain, facilitating modulation of Gal-3-induced bioactivity. Studies have shown that it is anti-oxidative, anti-inflammatory, cholesterol level-lowering, anti-bacterial, anti-cancer, and immunomodulatory, and reduces fibrosis in the kidney, liver, and adipose tissue. Modified citrus pectin (MCP) exerts its effects by inhibiting galectin-3, thereby downregulating the TLR4/MyD88/NF-κB signaling pathways implicated in Alzheimer’s disease. This mechanism contributes to the attenuation of neuroinflammation and oxidative stress, while concurrently promoting brain-derived neurotrophic factor (BDNF) expression, which facilitates neuronal plasticity and enhances cognitive performance [13,14,15].

Apart from anti-amyloid and anti-tau treatments, preclinical studies underline the benefits of antioxidant and anti-inflammatory treatments [16]. The low rates of Alzheimer’s disease in rheumatoid arthritis patients have led to the investigation of inflammatory pathways involved in the pathogenesis of Alzheimer’s disease. In particular, the emergence of the activities of activated microglia in neuroinflammation has accelerated Alzheimer’s research [17].

Alzheimer’s disease is the most common cause of dementia [18]. The effects of neuroinflammation and oxidative stress on cognitive processes must be identified, as well as the outcomes of preventative treatments, in order to make way for therapies that will stop the onset and progression of dementia [19]. This study investigates MCP’s effect on reducing neuroinflammation and its impact on cognitive function in an Alzheimer’s dementia model, focusing on its role in galectin-3 inhibition.

## 2. Materials and Methods

### 2.1. Animals and Experimental Design

This research was conducted after approval by the Afyonkocatepe Animal Experiments Local Ethics Committee, with the decision dated 8 July 2021, numbered 49533702/93. We used 6-week-old male Wistar Albino rats weighing 150–200 g. We procured the experimental animals and conducted the research at the Afyonkocatepe University Experimental Animal Center. We followed the ARRIVE guidelines (Animal Research: Reporting of In Vivo Experiments), a checklist of recommendations, while writing this study. We randomly selected rats and divided them into groups. After a one-week adaptation period, we randomly assigned the rats to four groups. We ensured that the rats had ad libitum access to standard laboratory pellets and were housed in standard cages under a temperature of 22–24 °C with constant humidity and a 12 h light–dark cycle. This study began after the rats had acclimated to the laboratory conditions for one week. We divided the rats into four groups, working with seven rats in each group. One rat died on the first day of scopolamine administration, so we proceeded with a control group of six rats. We decided on the MCP dose and duration based on Yin et al. (2020), who investigated the oral effectiveness of MCP in a diabetic Alzheimer’s model [20].

We determined the scopolamine dose for this study from two sources. Haider et al. (2016) demonstrated that scopolamine-induced neurochemical alterations and increased oxidative stress provide a robust model for dementia. Similarly, Hancianu et al. (2013) used scopolamine-induced dementia in rats to explore the neuroprotective effects of inhaled lavender oil and highlight the role of oxidative stress in the model. These methods offer valuable insights into mimicking dementia-related cognitive and neurochemical impairments. Therefore, we based our dementia induction methods on these approaches [21,22].

### 2.2. Study Groups

**Figure 1:**
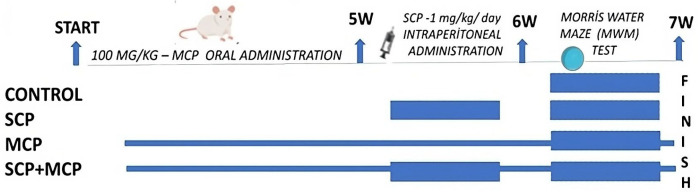
Experimental design.

The study design diagram is presented in Figure 1. The groups of rats used in the research are as follows.

Group 1, control group (CG): We followed the subjects for six weeks, and in the last week, we administered 0.9% saline solution intraperitoneally (i.p.) every day for seven days.

Group 2, scopolamine hydrobromide group (SCP): We followed the subjects for six weeks, and in the last week, we administrated 1 mg/kg scopolamine hydrobromide (Sigma, Taufkirchen, Germany), i.p., dissolved in 0.9% saline every day for seven days.

Group 3, scopolamine hydrobromide + modified citrus pectin (SCP+MCP): We added modified citrus pectin to the subjects’ drinking water at a rate of 100 mg/kg/day for six weeks. In the last week, we administered 1 mg/kg scopolamine (i.p.) dissolved in 0.9% saline solution for seven days.

Group 4, modified citrus pectin (MCP): We added modified citrus pectin to the subjects’ drinking water at 100 mg/kg/day for six weeks, and in the last week, we administered 0.9% saline solution (i.p.) for seven days.

Then, we performed the Morris water maze test (MWM) experiment to evaluate the cognitive functions of the rats.

### 2.3. The Morris Water Maze Test

The MWM test is a behavioral experiment used to assess spatial memory and learning in rodents. The test setup consists of a pool and a platform. The MWM test is conducted in two phases. The first phase, referred to as the pretest, involves training the subjects to climb onto the platform after being released into the pool. The second phase, known as the probe test, evaluates the time it takes for the subjects to locate the platform after it has been hidden. In this study, the pool used had a radius of 180 cm and a depth of 90 cm. During the experiments, the water temperature was maintained at a constant 26 °C. The platform in the maze was fixed in the western quadrant, and the training was conducted by releasing the rats from the northern, southern, and eastern directions. This procedure was repeated for 5 days, with three trials per day (Table 1). Each direction was marked using visual cues placed inside and outside the maze. During the pretest phase, the platform was positioned 2.5 cm above the water surface. The timer was started as soon as the rats were released into the water, facing the maze wall, and their latency to reach the platform was recorded using a stopwatch. The rats were allowed to remain in the water for up to 60 s or until they located the platform. If a rat failed to find the platform within this time, it was guided to the platform, where it was allowed to remain for 10 s. Afterward, the rat was dried with a towel. For each group, the second trial began immediately after all rats had completed the first trial. Twenty-four hours after the final trial on day 5, the probe test was conducted. In this phase, the pool was filled with additional tap water to submerge the platform just below the water surface. To conceal the platform and create uniform water opacity, coffee whitener was added to the pool. During this final test, the rats’ ability to swim directly to the platform’s location and their climbing times were recorded [23,24].

### 2.4. Sample Collection Process

**Figure 2:**
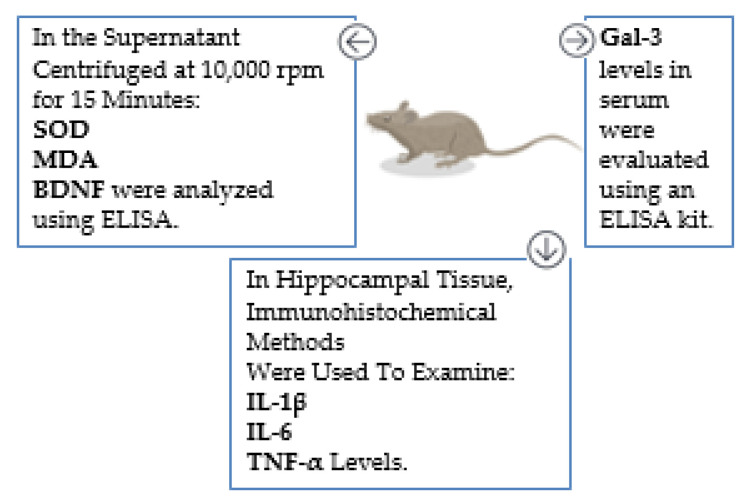
Representation of tests with a diagram.

Following the behavioral tests, the rats were sacrificed under ketamine/xylazine anesthesia on the last day. The blood of the rats was taken intracardially while under anesthesia. Serum samples were separated after centrifugation at 4000 rpm for 12 min and stored at −80 °C for measurement of galectin level. After the brain tissue was homogenized at 10,000 rpm for 15 min, the supernatant obtained was stored at −80°C for analysis of the superoxide dismutase (SOD) activity, malondialdehyde (MDA), and brain-derived neurotrophic factor (BDNF) levels. The hippocampal tissue samples were fixed in formalin and subsequently analyzed through immunohistochemical staining to assess the levels of IL-1β, IL-6, and TNF-α. On the day of the study, the separated serum and sample were brought to room temperature and processed following the procedures of the relevant kits (Figure 2). MDA, an important indicator of lipid peroxidation; SOD, an important indicator of antioxidant defense; and BDNF, which plays a critical role in neurological recovery and repair, were preferred because they are important biomarkers; many studies have been conducted on them; and they are frequently evaluated in studies related to neurological diseases, especially Alzheimer’s.

#### 2.4.1. Enzyme-Linked Immunosorbent Assay (ELISA) Tests

We prepared the supernatant for antioxidant studies after homogenizing the brain tissue at 10,000 rpm for 15 min. We used ELISA kits to measure MDA, BDNF, SOD, and Gal-3.

#### 2.4.2. Measurement of Tissue MDA Levels

We performed MDA measurement in tissue using the SunRed brand Rat MDA ELISA measurement kit (Catalogue No: 201-11-0157) (Shanghai Sunred Biological Technology Co., Ltd., Shanghai, China). We read absorbance using the Chromate 4300 brand ELISA reader (Awareness Technology, Inc., Martin Hwy, Palm City, FL, USA). The measurement was made at 450 nm. We calculated the data using a linear regression analysis, and the results are expressed in nmol/L.

#### 2.4.3. Measurement of Tissue SOD Levels

We performed the SOD measurement in tissue using the SunRed brand Rat SOD ELISA measurement kit (Catalogue No: 201-11-0169) (Shanghai Sunred Biological Technology Co., Ltd., Shanghai, China). We read absorbance using the Chromate 4300 brand ELISA reader (Awareness Technology, Inc., Martin Hwy, Palm City, FL, USA). The measurement was made at 450 nm. We calculated the data using a linear regression analysis, and the results are expressed in ng/mL.

#### 2.4.4. Measurement of Tissue BDNF Levels

We measured BDNF in tissue using the SunRed brand Rat BDNF ELISA measurement kit (Catalogue No: 201-11-0477) (Shanghai Sunred Biological Technology Co., Ltd., Shanghai, China). We performed the absorbance reading on the Chromate 4300 ELISA reader device (Awareness Technology, Inc., Martin Hwy, Palm City, FL, USA) at 450 nm. We calculated the data using a linear regression analysis and expressed the results as ng/mL.

#### 2.4.5. Measurement of Serum Gal-3 Levels

We performed the Gal-3 measurement in serum using the USCN brand Rat Gal-3 ELISA measurement kit (Catalogue No: SEA303Ra) (Shanghai Sunred Biological Technology Co., Ltd., Shanghai, China). We conducted the absorbance reading on the Chromate 4300 ELISA reader device (Awareness Technology, Inc., Martin Hwy, Palm City, FL, USA) at 450 nm. We calculated the data using a 4-parameter logistic regression analysis and expressed the results as ng/mL.

#### 2.4.6. Immunohistochemistry Test

The part of the hippocampus that plays a major role in spatial memory is called “location cells”. These are a type of pyramidal neurons primarily found in the CA3 and CA1 regions of the hippocampus. For this study, we used histopathological data from the CA3 and CA2 regions of the hippocampus [25]. After obtaining 5-micron sections from paraffin tissue blocks, we performed deparaffinization and clarified the tissue with xylene. Immunohistochemistry (IHC) staining was then conducted on the paraffin sections of the hippocampal area. We determined the levels of IL-1β, IL-6, and TNF-α immunohistochemically using the H-SCORE method. TNF-α was used at a 1/100 dilution, IL-1β at a 1/250 dilution, and IL-6 at a 1/100 dilution as primary antibodies. We used HRP Goat Anti-Mouse at a 1/250 dilution as the secondary antibody.

After preparing the tissue sections for staining, we heated them in a microwave for 20 min in citrate buffer for antigen retrieval. We then assessed endogenous peroxidase activity with 3% H_2_O_2_. After washing with PBS buffer, we blocked the proteins for 25 min with a blocking solution and incubated the tissue with the primary antibodies (IL-1β, IL-6, and TNF-α) at +4 °C overnight. Following a PBS wash, we treated the sections with Avidin and Biotin solutions for 30 min each. We applied an AEC single solution and left it at room temperature for 10–12 min. Counterstaining was performed by placing the sections in Mayers Hematoxylin for 2 min, and they were evaluated under a light microscope after covering. The results were quantitatively obtained using the H-SCORE method. The H-score is used to assess the extent of nuclear immunoreactivity and is applicable to steroid receptors. The score is calculated using the formula 3 × percentage of strongly staining nuclei + 2 × percentage of moderately staining nuclei + percentage of weakly staining nuclei, yielding a range from 0 to 300.

For the detection of cytokine levels, we used the following primary antibodies: anti-IL-6 (catalog no: A11115, Abclonal, Woburn, MA, USA), anti-TNF-α (catalog no: A11534, Abclonal), and anti-IL-1β (catalog no: SC-52012, Santa Cruz Biotechnology, Dallas, TX, USA). All antibodies were diluted at 1:250 for optimal performance. We used an HRP-based detection kit (Thermo Fisher TP-126-H2, Waltham, MA, USA) was utilized, which included a ready-to-use blocking solution as part of the kit. These reagents were selected for their proven specificity and reliability in immunological assays, ensuring accurate detection and quantification of the targeted cytokines [26,27].

#### 2.4.7. Modified Citrus Pectin (MCP)

Modified citrus pectin (Pectasol^®^Modified Citrus Pectin Powder, EcoNugenics, 16 oz, 454 gr, Santa Rosa, CA, USA), a form of pectin that has been chemically modified for better absorption in the digestive tract, contains carbohydrates (4 g), dietary fiber (3 g), sodium (160 mg), potassium (420 mg), and modified citrus pectin powder (5 g) [28]. PectaSol^®^ contains modified citrus pectin (MCP) made from citrus peels, specifically lemons, limes, and oranges. The modification method uses enzymatic hydrolysis and pH modifications to break down complex pectin molecules into smaller, more accessible fragments, increasing absorption and effectiveness in detoxifying and immunological health. Enzymes such as endo-polygalacturonase are used to lower the molecular weight of pectin and improve its functional characteristics [29].

### 2.5. Statistical Analysis

The Statistical Package for the Social Sciences (SPSS, version 20.0; IBM Corp., 2019, Armonk, NY, USA) was used to analyze the data obtained from the research. All data are presented as mean ± standard error of the mean (SEM) on a group basis. To compare groups, one-way ANOVA (post hoc LSD) was performed. A paired sample *t*-test was used to compare baseline and final weight changes. Additionally, Pearson’s r was calculated to determine the relationship between variables. A *p*-value of < 0.05 was considered statistically significant.

## 3. Results

### 3.1. Body Weights

There was no significant difference in body weights between groups before and after the experiments (*p* > 0.05), nor in weight changes within groups over time (*p* > 0.05).

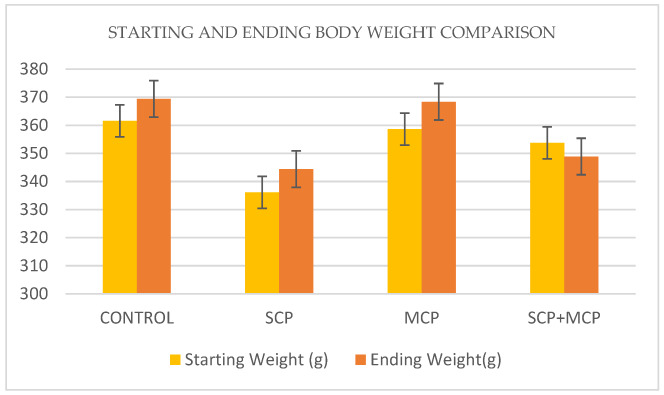



### 3.2. MDA, SOD, BDNF, and Plasma Galectin-3 Levels in Hippocampus Tissue

**Figure 3:**
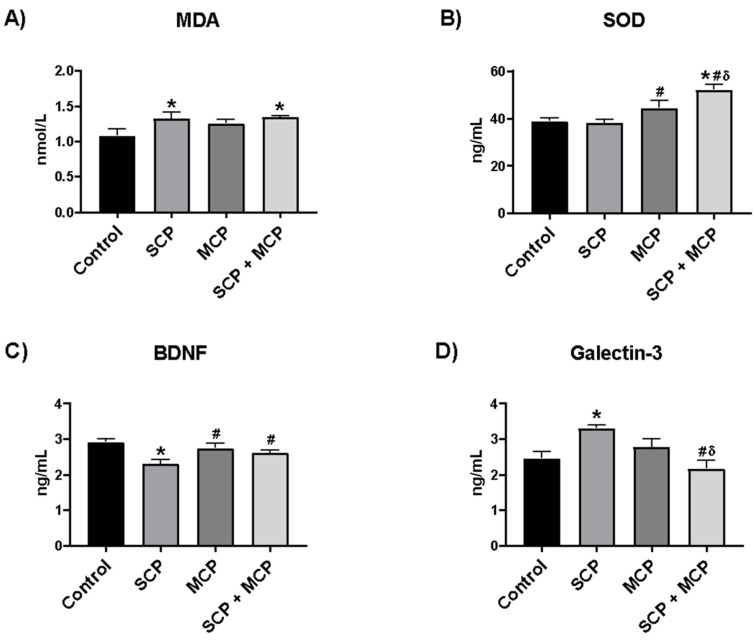
The changes in the hippocampal malondialdehyde, MDA (**A**); superoxide dismutase, SOD (**B**); brain-derived neurotrophic factor, BDNF (**C**); and plasma galectin-3 levels (**D**) in the control (*n* = 6), scopolamine (SCP, *n* = 7), modified citrus pectin (MCP, *n* = 7), and SCP+MCP (*n* = 7) groups. Data are presented as mean ± standard error of the mean (SEM). * *p* < 0.05 compared to the control group. # *p* < 0.05 compared to the SCP group. δ *p* < 0.05 compared to the MCP group.

The hippocampal MDA level was significantly higher in the SCP and SCP+MCP groups than in the control group (*p* = 0.027, *p* = 0.017, respectively) (Figure 3A). Hippocampal SOD level was higher in the SCP+MCP group compared to all other groups (control, SCP, and MCP) (*p* < 0.001, *p* < 0.001, *p* = 0.02, respectively). Additionally, SOD was found to be higher in the MCP group compared to the SCP group (*p* = 0.049). In the SCP group, the hippocampal SOD level was lower than in the control group, although it was not significant (*p* > 0.05). (Figure 3B). BDNF was significantly lower in the SCP group compared to the control group (*p* = 0.001) and higher in the MCP and SCP+MCP groups compared to the SCP group (*p* = 0.005, *p* = 0.043, respectively) (Figure 3C). Plasma galectin-3 level was significantly higher in the SCP group compared to the control group (*p* = 0.007) and lower in the SCP+MCP group compared to the SCP and MCP groups (*p* < 0.001, *p* = 0.036, respectively) (Figure 3D).

### 3.3. TNF-α, IL- 1β, and IL-6 Immunoreactivity in the Hippocampus

**Figure 4:**
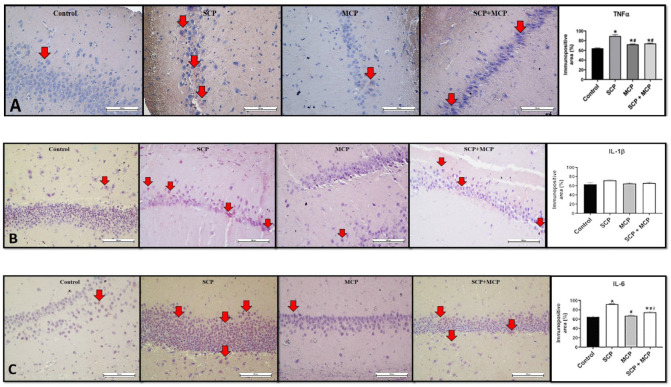
Representative immunohistochemical staining micrographs and the alterations in the immunopositive area of tumor necrosis factor alpha, TNF-α (**A**); interleukin-1 beta, IL-1β (**B**); and interleukin-6, IL-6 (**C**), in the control (*n* = 6), scopolamine (SCP, *n* = 7), modified citrus pectin (MCP, *n* = 7), and SCP+MCP (*n* = 7) groups. Data are presented as mean ± standard error of the mean (SEM). * *p* < 0.05 compared to the control group; # *p* < 0.05 compared to the SCP group; δ *p* < 0.05 compared to the MCP group.

TNF-α immunoreactivity was significantly higher in all experimental groups (SCP, MCP, and SCP+MCP) compared to the control group (*p* < 0.001, *p* = 0.004, and *p* = 0.001, respectively). However, it was significantly lower in the MCP and SCP+MCP groups compared to the SCP group (*p* < 0.001 and *p* < 0.001, respectively) (Figure 4A). There was no significant difference between the groups regarding IL-1β immunoreactivity (*p* > 0.05) (Figure 4B).

IL-6 immunoreactivity was significantly elevated in the SCP and SCP+MCP groups compared to the control group (*p* < 0.001 and p=0.001, respectively). However, it was significantly reduced in the MCP and SCP+MCP groups compared to the SCP group (*p* < 0.001 and *p* < 0.001, respectively).

Additionally, IL-6 immunoreactivity was significantly higher in the SCP+MCP group compared to the MCP group (*p* = 0.004) (Figure 4C).

### 3.4. Morris Water Maze (MWM) Training

**Figure 5:**
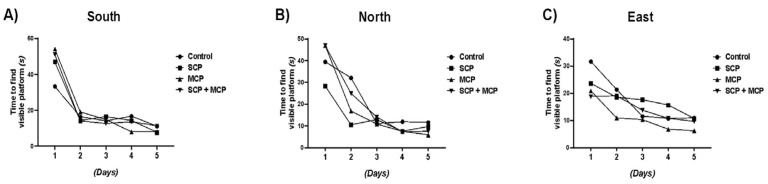
Morris water maze training days 1–5; Figures (**A**–**C**) show the results of the Morris water maze pretest conducted in the South (**A**), North (**B**), and East (**C**) regions. X-axis (days); Y-axis (time to find the platform); control (*n* = 6), scopolamine (SCP, *n* = 7), modified citrus pectin (MCP, *n* = 7), and SCP + MCP (*n* = 7) groups. During the MWM test (days 1–5), the time to find the platform gradually decreased in all directions (south, north, and east). Data are presented as mean values.

The decrease in times in all groups in the WMW test indicates that learning occurred and the subjects adapted to the task (Figure 5).

For the east direction, the SCP group showed longer times, while the MCP group showed shorter times compared to the control (*p* < 0.001, *p* = 0.021). Differences in escape time may be related to subjects being better or worse at using environmental cues in certain directions.

**Figure 6:**
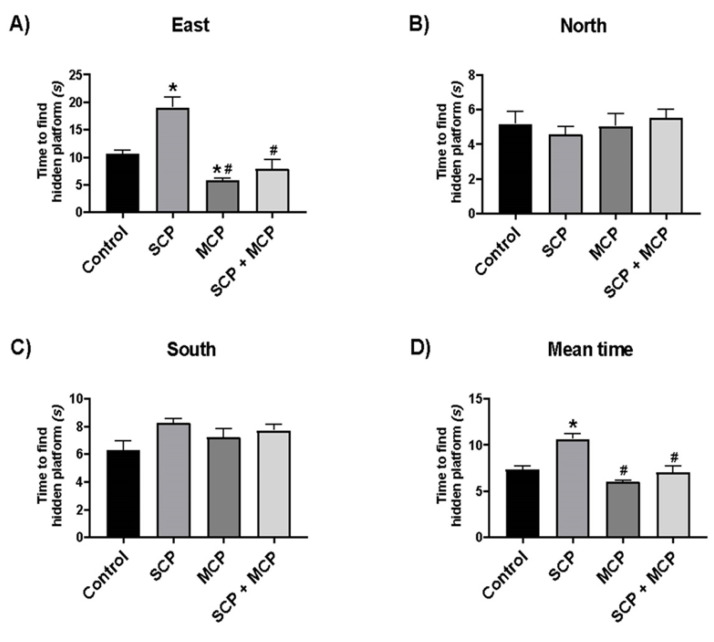
Morris water maze test results for directions East (**A**), north (**B**), south (**C**), and Probe Test mean time (**D**) in the control (*n* = 6), scopolamine (SCP, *n* = 7), modified citrus pectin (MCP, *n* = 7), and SCP+MCP (*n* = 7) groups. Data are presented as mean ± standard error of the mean (SEM). * *p* < 0.05 compared to the control group. # *p* < 0.05 compared to the SCP group.

Additionally, the time to find the hidden platform was shorter in the MCP and SCP+MCP groups compared to the SCP group (*p* < 0.001 and *p* < 0.001, respectively) (Figure 6A). There was no difference between the groups in terms of the time to find the hidden platform for the north and south directions (Figure 6B,C). When the average time of the rats to find the hidden platform was compared, it was seen that the mean time was longer in the SCP group compared to the control group (*p* < 0.001), and shorter in the MCP and SCP+MCP groups compared to the SCP group (*p* < 0.001 and *p* < 0.001, respectively) (Figure 6D).

Furthermore, it was found that mean time is negatively correlated with BDNF and positively correlated with Gal-3, TNF-α, IL-1β, and IL-6 (r = −0.391, *p* = 0.044; r = 0.446, *p* = 0.02; r = 0.672, *p* = 0.012; r = 0.61, *p* = 0.027; and r = 0.0796, *p* = 0.002, respectively). Furthermore, there was a significant negative correlation between BDNF and TNF-α, as well as BDNF and IL-1β (r = −0.76, *p* = 0.003; r = −0.59, *p* = 0.034). Additionally, a negative correlation was observed between Gal-3 and SOD (r = −0.594, *p* = 0.001).

In summary, SCP application increased the levels of lipid peroxidation product MDA and the inflammation markers TNF-α, IL-6, and plasma galectin-3 while decreasing the levels of BDNF, a neurotrophic factor. In addition, a longer time taken to find the hidden platform in the MWM test indicates that SCP causes impairment in spatial memory. In contrast, the application of MCP and SCP decreased TNF-α, IL-6, and Gal-3 while increasing BDNF and ameliorating the impairment in spatial memory. The co-administration of SCP and MCP was also found to be effective in increasing the antioxidant enzyme SOD (Table 2).

## 4. Discussion

These results demonstrate that MCP is effective in both suppressing inflammation, particularly through the inhibition of galectin-3, and supporting cognitive functions through neuroprotective effects. Gal-3 is a protein that belongs to the galectin family, a group of proteins that bind carbohydrates. The basic processes of cell adhesion, inflammation, immunological response, and the development of cancer are all impacted by Gal-3. It is well known for interacting with various molecules and controlling cell signaling pathways [30]. A meta-analysis study, including 17 studies and examining a total of 905 patients, shows that Gal-3 levels are high in Alzheimer’s disease patients [31]. Similar positive correlations between Gal3 and cognitive impairment have been observed in investigations [32].

Amyloid-β (Aβ) peptide and tau protein are primary components of plaques and tangles, respectively, and are considered significant targets of focus in the development of treatments [33]. Our results support earlier research showing a negative correlation between Gal-3 and SOD. The findings suggest that SCP and MCP together produce a synergistic effect rather than MCP mitigating the impairments caused by SCP in rats. This highlights the enhanced impact of their combined action compared to their individual effects. Galectin-3 is a proinflammatory protein expressed and released by active microglia in the damaged brain region. As seen in the research findings, the high SOD and low Gal-3 levels in the groups where MCP and SCP were administered together, unlike the groups that received only MCP, suggest that the effect will be better revealed on the basis of inflammation. Therefore, it can be said that galectin, a protein involved in regulating the inflammatory process and easily detected in serum, and treatments that antagonize it hold significant hope for treating Alzheimer’s disease [34].

Citrus pectin (CP), a pectin derivative, is a complex polysaccharide with an anionic structure that can be obtained from the white peel and flesh of many citrus fruits such as lemon, orange, grapefruit, and apple. Pectins with large and complex structures containing varying degrees of esterification are converted into MCP with a low molecular weight of 15 kilodaltons (kDa) and an esterification degree of less than 5% by a specific pH- and temperature-controlled enzymatic processes. This form has higher bioavailability and bioactivity. MCP has been extensively studied as a Gal-3 inhibitor in numerous cell and animal models [35].

Since pectin is known to give a feeling of satiety and limit calorie intake due to its fiber and protein content in animal studies, weight was also monitored in this study, but no significant difference was determined [36].

In this study, scopolamine was used to induce an experimental model of amnesia due to its antimuscarinic properties that elevate oxidative stress and neuroinflammation, thereby leading to neuronal damage and mimicking neurodegeneration [37]. Neurological behavior defects are caused by neuronal apoptosis generated by reactive oxygen species (ROS). Metalloenzymes called superoxide dismutases (SODs) are essential in the body’s antioxidant defense against oxidative stress. SOD plays a crucial part in preserving redox equilibrium by converting superoxide radicals to hydrogen peroxide. It is known that SOD is found in the hippocampus, striatum, suprachiasmatic nucleus, glial cells, and cerebrospinal fluid and is associated with neurological disorders such as learning and memory, cerebral ischemia, and traumatic brain injury. Murakami et al. [38] determined that agents that promote the activation of SOD may be a therapeutic strategy for the inhibition of Alzheimer’s disease progression [39,40].

Proinflammatory cytokines (TNF-α, IL-1β, and IL-6) play a significant role in the progression and expansion of neuro-inflammation. Many studies supporting the relationship between Alzheimer’s disease and inflammatory agents have been found noteworthy, as they have the potential as biomarkers for early therapeutic interventions. In particular, the significant relationships found between the polymorphisms identified in these inflammatory agents and Alzheimer’s disease support this theory [11,41]. One of the important results of this study is the significant decrease in TNF-α and IL-6 levels in the hippocampus tissue in the MCP group. Our research findings indicate that MCP may effectively mitigate the inflammatory response generated by scopolamine, while demonstrating that IL-1β is less efficacious in this context, alongside the dynamic interactions of TNF-α and IL-6 regarding the inflammatory response. The lack of elevation of IL-1β suggests that further investigation is required to understand the complexity of the inflammatory response induced by scopolamine and the different interactions between the components of this response. This study shows that MCP, an antigalectin-3 inhibitor, has an ameliorative effect on dementia, supporting research that galectin-3 regulates inflammation and affects cognitive performance through inflammatory activity.

Galectin-3 is a proinflammatory protein expressed and released by active microglia in the damaged brain region. As seen in the research findings, the high SOD and low Gal-3 levels in the groups where MCP and SCP were administered together, unlike the groups that received only MCP, suggest that the effect will be better revealed on the basis of inflammation. Information on MCP, IL-6, and inflammation mechanisms has been widely discussed in the scientific literature. MCP plays a role in immune system regulation by exhibiting anti-inflammatory effects. In addition, it has been shown that it can regulate the release of inflammatory cytokines such as IL-6. In particular, it has been reported that MCP is effective in controlling inflammation and can regulate inflammatory processes by reducing oxidative stress and suppressing inflammatory signaling pathways such as NF-κB. Such biological peptides (bioactive peptides, BAPs) can reduce the levels of cytokines such as IL-6 and TNF-α or limit their effects. The potential of MCP to limit IL-6 production by regulating the activity of NF-κB highlights the therapeutic benefits of MCP in inflammatory diseases. Gal-3 is an important regulator of inflammatory processes and is known to trigger inflammation via the TLR4/MyD88/NF-κB (Toll-Like Receptor 4/Myeloid differentiation primary response 88/Nuclear Factor kappa-light-chain-enhancer of activated B cell) pathway. However, IL-1β production is more specifically activated via the NLRP3 inflammasome and caspase-1. While MCP suppresses TLR4/MyD88/NF-κB signaling as a Gal-3 inhibitor, contributing to the decrease in cytokines such as IL-6 and TNF-α, its limited effect on the NLRP3 inflammasome may explain the lack of change in IL-1β. It can be said that it has a limited interaction with IL-1β but may be effective in suppressing the general inflammatory response [42].

Although MCP suppresses inflammation by inhibiting Gal-3, some studies have shown that low levels of Gal-3 may produce proinflammatory effects in certain contexts [43].

It is possible that MCP increased TNF-α and IL-6 by affecting other proinflammatory mechanisms such as the TLR4/MyD88/NF-κB pathways in addition to Gal-3 inhibition.

In addition to the anti-inflammatory effects of MCP, it is possible that it induces a short-term proinflammatory response during the initial application. This may occur during the reorganization process of immune cells. MCP has been reported to switch macrophages from the M1 (proinflammatory) to M2 (anti-inflammatory) phenotype. However, a transient increase in inflammatory cytokines may be observed during the transition process [44].

Local immune cells, called phagocytic cells, distributed in many parts of the brain, release BDNF responsible for memory and synaptic regulation in neuronal plasticity. BDNF is a protein known to be associated with cognitive functions. An essential neurotrophic factor, BDNF, is expressed in the brainstem, hypothalamus, cortex, and hippocampus. It promotes synaptic activity, brain cell survival, and the decrease in neuroinflammation. The fact that beta-amyloid formation reduces BDNF shows that BDNF has the potential to be an effective pathway in correcting cognitive impairments such as dementia in Alzheimer’s disease. As the BDNF level increases, the average time it takes for rats to find the hidden platform decreases, supporting the BDNF–dementia association in the literature [45]. The inverse correlation between BDNF and inflammation markers and the increased BDNF in the MCP group supported by the results of this study show that MCP may be an essential potential agent for use in the treatment of Alzheimer’s disease. MCP’s antioxidant and anti-inflammatory properties may positively affect neuronal survival and cognitive function by increasing BDNF levels.

The MWM test was used to evaluate hippocampal activities. Our research results show that applying MCP improved spatial memory impairment. Rats in both the MCP and SCP+MCP groups took significantly shorter time to find the hidden platform than those in the SCP group. These findings suggest that MCP has the potential to alleviate scopolamine-induced cognitive impairments. Treatments that reduce oxidative stress and neuroinflammation to prevent neurotoxicity may halt or reduce the progression of dementia. We think that the effect of MCP on dementia and its potential as a primary treatment alternative is worth investigating [46].

This research is the first to examine the effect of MCP on cognitive functions in an Alzheimer’s type dementia model. Our present study showed that MCP reduces inflammatory markers and gal-3, has antioxidant activity, and improves memory functions in an experimental model. The anti-inflammatory and antioxidant findings are also consistent with in vivo and in vitro studies conducted with MCP [47].

Hippocampal malondialdehyde (MDA) levels were significantly higher in both the SCP and SCP+MCP groups compared to the control group, indicating that cellular damage and oxidative stress caused by scopolamine were at a high level. The fact that MDA levels remained high despite the administration of MCP may be explained by the severity of oxidative stress caused by scopolamine, inadequate dose of MCP, or timing-related factors. Galectin-3, inhibited by MCP, displays protective mitochondrial roles and its suppression may have increased tissue damage by disrupting redox homeostasis [48]. It indicates that additional studies are needed to better understand the effectiveness of MCP on lipid peroxidation. A limitation of this study is that different experimental protocols were not used to demonstrate memory functions. Additionally, the different effects of MCPs have not been fully evaluated, and further research is needed to elucidate these pathways. To clarify the mechanisms of anti-galectin-3 therapies in neurodegenerative illnesses, more research is required.

## 5. Conclusions

The study’s findings show that MCP has antioxidant activity, lowers inflammatory markers and Gal-3 levels, enhances cognitive skills in the Alzheimer’s dementia model, and generally improves memory functions. These benefits of MCP suggest that it deserves consideration as a possible therapeutic alternative for Alzheimer’s disease. Although research results indicate that MCP may be promising in the treatment of cognitive problems, research is needed to support its effectiveness with pathophysiological mechanisms.

## Figures and Tables

**Table 1:** The order of locations where mice were released in the pre-test and probe test.

	Pre-Test					Probe Test		
	Day 1	Day 2	Day 3	Day 4	Day 5	Day 6		
Trial 1	S	N	E	S	N	E	S	N
Trial 2	N	E	S	N	E	S	N	E
Trial 3	E	S	N	E	S	N	E	S
Platform location	W	W	W	W	W	No platform		

**Table 2:** This table presents the correlation coefficients (r values) and their corresponding *p*-values for various biomarkers (BDNF, Gal-3, TNF-α, IL-1β, IL-6, and SOD) with the mean time to find the hidden platform in the Morris water maze (MWM) test. The correlations were analyzed to understand the relationships between spatial memory performance (mean time) and the levels of these biomarkers, which are related to inflammation and antioxidant activity.

Correlation	r Value	*p* Value
Mean time and BDNF	−0.391	0.044
Mean time and Gal-3	0.446	0.020
Mean time and TNF-α	0.672	0.012
Mean time and IL-1β	0.610	0.027
Mean time and IL-6	0.796	0.002
BDNF and TNF-α	−0.760	0.003
BDNF and IL-1β	−0.590	0.034
Gal-3 and SOD	−0.594	0.001

## Data Availability

All data included in this study are available upon request by contact with the corresponding author.
